# High durability and stability of 2D nanofluidic devices for long-term single-molecule sensing

**DOI:** 10.1038/s41699-023-00373-5

**Published:** 2023-02-23

**Authors:** Mukeshchand Thakur, Nianduo Cai, Miao Zhang, Yunfei Teng, Andrey Chernev, Mukesh Tripathi, Yanfei Zhao, Michal Macha, Farida Elharouni, Martina Lihter, Liping Wen, Andras Kis, Aleksandra Radenovic

**Affiliations:** 1 Laboratory of Nanoscale Biology, Institute of Bioengineering, School of Engineering, EPFL, 1015 Lausanne, Switzerland; 2 CAS Key Laboratory of Bio-Inspired Materials and Interfacial Science, Technical Institute of Physics and Chemistry, Chinese Academy of Sciences, 100190 Beijing, China; 3 School of Future Technology, University of Chinese Academy of Sciences, 100049 Beijing, China; 4 Laboratory of Nanoscale Electronics and Structure, Institute of Electrical Engineering and Institute of Materials Science and Engineering, School of Engineering, EPFL, 1015 Lausanne, Switzerland

**Keywords:** Nanopores, Two-dimensional materials

## Abstract

Nanopores in two-dimensional (2D) membranes hold immense potential in single-molecule sensing, osmotic power generation, and information storage. Recent advances in 2D nanopores, especially on single-layer MoS_2_, focus on the scalable growth and manufacturing of nanopore devices. However, there still remains a bottleneck in controlling the nanopore stability in atomically thin membranes. Here, we evaluate the major factors responsible for the instability of the monolayer MoS_2_ nanopores. We identify chemical oxidation and delamination of monolayers from their underlying substrates as the major reasons for the instability of MoS_2_ nanopores. Surface modification of the substrate and reducing the oxygen from the measurement solution improves nanopore stability and dramatically increases their shelf-life. Understanding nanopore growth and stability can provide insights into controlling the pore size, shape and can enable long-term measurements with a high signal-to-noise ratio and engineering durable nanopore devices.

## Introduction

Nanopores in two-dimensional (2D) materials are a promising class of solid-state sensors and serve as a versatile tool for mimicking biological pores and channels in cells^1–5^. Most commonly studied 2D materials for nanopores are graphene^2,6–8^, MoS_2_^9–12^, WS_2_^13,14^, hBN^15^, and more recently MXenes^2,16,17^. A typical 2D nanopore device consists of a nanopore in a free-standing atomically thin membrane over a supporting aperture that separates two reservoirs. Electrically charged biopolymers such as DNA, RNA, or proteins are driven through the nanopore under an applied electrical field and generate distinct signals in ionic current that are characteristic of translocating molecules. The 2D nanopore devices have become an important tool for studying single-molecule biophysics, ion transport, and selectivity.

Solid-state nanopores in general, have inspired many novel applications such as water desalination^18,19^, solute and gas separation^14,20^, osmotic energy^3^, and digital DNA readout^21^. Of all the variety of 2D nanopores reported so far, nanopores in monolayer MoS_2_ membranes have gained considerable attention, especially in biosensing applications. An ultrathin tri-atomic monolayer MoS_2_ (~0.65 nm), in principle, provides high spatial resolution approaching the physical distance of two adjacent DNA bases (~0.34 nm). Compared to 2D graphene membranes, the sticking of DNA bases to the MoS_2_ is relatively weak^22^, which makes it a lucrative tool to study at a single molecular level. Indeed, MoS_2_ nanopores have been shown to detect DNA molecules down to single-nucleotide resolution^23^ and even differentiate topological variations on DNA^24^. Recently, Graf et al.^25^ demonstrated the fabrication of a MoS_2_ nanopore field-effect transistor capable of detecting DNA simultaneously in ionic as well as in transverse channel through MoS_2_ featuring the versatility of 2D MoS_2_ nanopores in different sensor modalities. Currently, the solid-state nanopore technology is still limited to lab-scale research due to practical bottlenecks that hinder its commercial application^2,26–28^.

The device yield, variability, stability, and reliability are important performance metrics for solid-state sensors^26,27^. Merchant et al.^6^ deposited a thin TiO_2_ layer (~5 nm) on the graphene membrane to address the issue of noise and robustness of the nanopore device. Although the devices showed improved noise compared to the undeposited counterpart, the coating increased the overall thickness of the membrane. Unfortunately, the stability of 2D nanopore devices has been poorly studied and thus needs to be addressed to realize their commercial potential as sensors. Fortunately, few groups have studied and tried to address the stability of silicon-based solid-state nanopore devices^13,29–31^. Progress in the growth of high-quality MoS_2_, large-area wafer-scale substrate fabrication, and transfer has improved the scalability and efficiency of MoS_2_ nanopore device fabrication^32,33^.

Nevertheless, further challenges need to be addressed for the development of 2D nanopore devices as biosensors. Oxidation of 2D materials has been a major challenge toward the use of 2D materials as biosensors. Gao et al.^34^ observed morphological changes such as monolayer cracking and oxidation along the grain boundaries in CVD-grown MoS_2_ and WS_2_ monolayers upon exposure to air. Further studies have shown that upon exposure to air under ambient conditions, oxygen atoms spontaneously incorporate in 2D MoS_2_ layers^35^ and contribute to poor air stability or limit the use of 2D materials in ambient conditions. Voltage-mediated delamination of 2D monolayers has been observed during ion-transport measurements in atomically thin membranes^36^. Such damage is inevitable as 2D nanopore sensors are often exposed to air while device fabrication and the experimental setup require exposure aqueous solution. Thus oxidation of the 2D material^34,35,37,38^, and nanopore expansion in standard experimental conditions need detailed examination^27^ as these parameters are critical for the development of 2D nanopore devices as well as for the advancement of 2D materials research in general. To address these challenges, in this paper we investigate and discuss major reasons for the instability of monolayer 2D MoS_2_ membranes and their nanopores, which renders low yield, reliability, and device failure. We observe that the delamination of the monolayer MoS_2_ from its substrate is one the main reason for the instability of nanopore devices. By increasing the hydrophobicity of the SiN_x_ substrate by an organosilicon coating prior to transferring MoS_2_ strengthens MoS_2_-SiN_x_ interfacial interaction, improves adhesion, and thereby reduces detachment from the substrate. Furthermore, we also found that the chemical oxidation of the MoS_2_ monolayer creates and enlarges the defects in the membrane_,_ leading to pore enlargement in an aqueous solution. We show that reducing the oxygen concentration level in the experimental buffer improves the nanopore lifetime by slowing down the pore edge dissolution. Reinforcing MoS_2_-SiN_x_ interaction and minimizing the MoS_2_ oxidation process in the experimental aqueous facilitates continuous long-time DNA sensing on the same pore (>3 h). Finally, we discuss and provide guidelines to address other phenomena that can potentially compromise 2D nanopore devices such as nanopore clogging, surface hydrocarbon contamination, and electrostatic membrane damage that routinely lead to device failure.

## Results and discussion

### 2D MoS_2_ nanopore: device architecture and nanopore instability

**Fig. 1:**
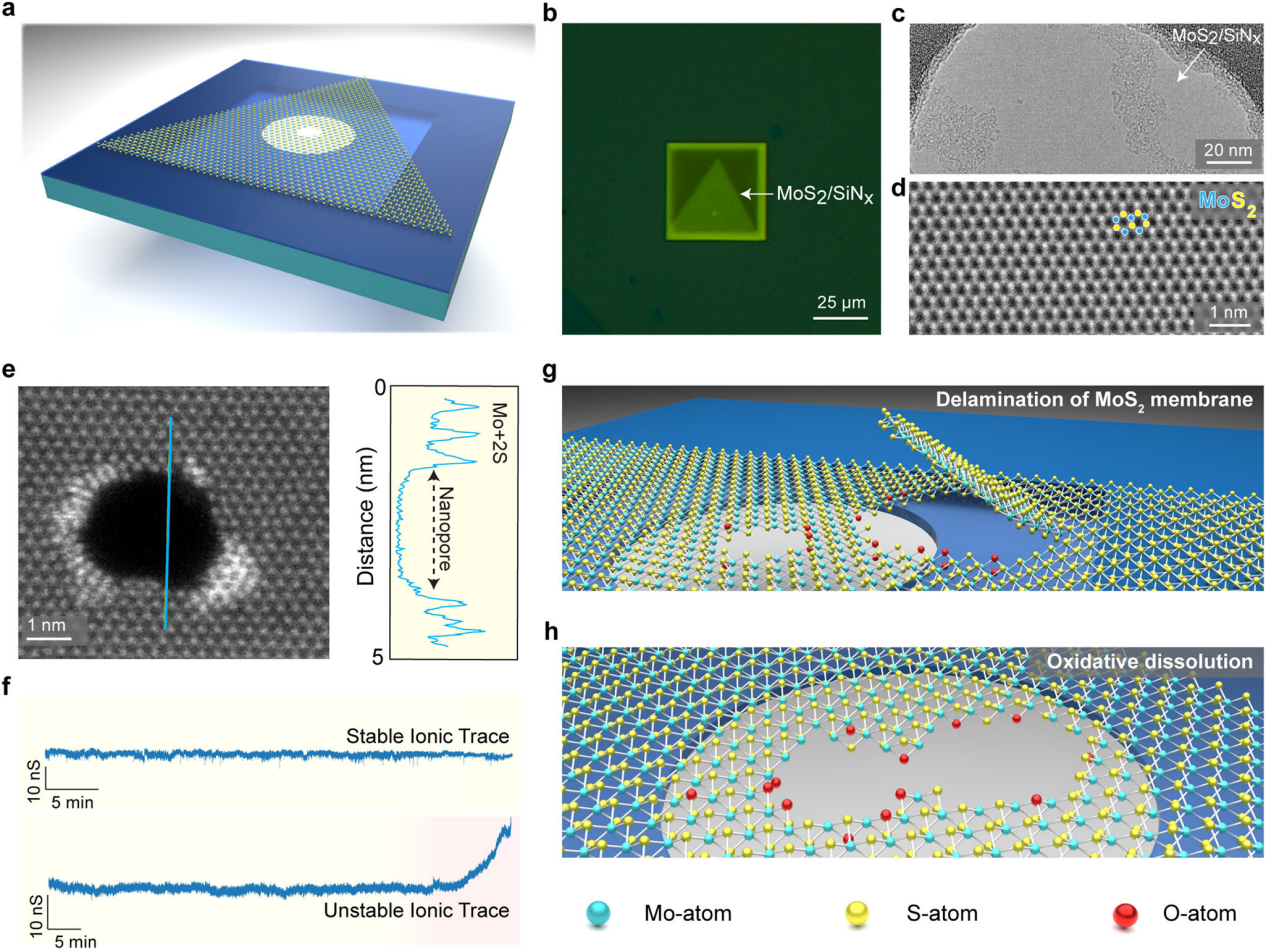
A 2D MoS_2_ nanopore and nanopore instability. **a** Schematic showing a single crystal of monolayer MoS_2_ transferred over a SiN_x_ membrane (~20 nm thick). The crystalline monolayer MoS_2_ is free-standing over a SiN_x_ aperture of ~80 nm. A suitable nanopore is then created in the suspended part. **b** Optical micrograph of a 2D nanopore device after transfer of a monolayer MoS_2_. **c** The bright-field TEM image of a clean suspended MoS_2_ membrane and (**d**) an aberration-corrected ADF-STEM image of the membrane show a perfect lattice with brighter Mo-atoms (indicated with blue circles) and relatively lighter S-atoms (indicated with yellow circles). **e** A nanopore (~2.5 nm) drilled in ADF-STEM mode is shown with an intensity profile highlighting the Mo-atoms with a dangling bond at the edge of the nanopore. **f** Representative ionic current traces of two different nanopore devices that show stable and increasing open-pore current with time, respectively, emphasize the instability in 2D nanopores. **g**, **h** Schematic showing the mechanisms of device instability issues arising during the course of a nanopore experiment. The red spheres represent oxygen atoms at the edges of the MoS_2_ nanopore.

A typical MoS_2_ nanopore device comprises a suspended 2D material over a thin SiN_x_ substrate (Fig. 1a). The SiN_x_ membrane (~30 × 30-μm-square) is about 20 nm thick and consists of an aperture of 80–100 nm in diameter defined by e-beam lithography (Supplementary Figs. 1, 2)^9,33^. A monolayer of MoS_2_ is then deterministically transferred^9^ to the membrane (Fig. 1b) such that there is a free-standing MoS_2_ over the aperture (Fig. 1c). See the Materials and Methods section for monolayer MoS_2_ growth and transfer.

A 2D MoS_2_ membrane is an atomically thin transition metal dichalcogenide that comprises two hexagonal planes of S-atoms and a hexagonal plane of Mo-atoms as seen in the aberration-corrected ADF-STEM image (Fig. 1d). In a monolayer form, the Mo-atom is covalently attached to the S-atoms in a trigonal prismatic geometry^39^. A nanopore is formed in the monolayer using either TEM-based method^9,12^ or in situ via the electrochemical-reaction (ECR) method^39^. Figure 1e shows a single MoS_2_ nanopore in a monolayer with an approximate diameter (d_TEM_) of ~2.5 nm drilled using STEM at 80 kV^9^. The nanopore in the monolayer MoS_2_ shows edges terminated with mainly Mo-atoms. The brighter Mo-atoms are due to the heavier atom contrast of the Mo-atom compared to the S-atom. The nanopore device is then assembled into a custom-built flow cell^9^ filled with an electrolyte (1 M KCl), and the ionic current through the nanopore is measured by applying a voltage across the pore. Figure 1f shows an example ionic current time trace from two representative MoS_2_ nanopore devices with stable (at 200 mV) and unstable current trace (300 mV) in 1 M KCl acquired with a 10 kHz filter and 100 kHz sampling rate. It must be noted that the instability can also occur within a few minutes after pore wetting causing a larger open pore current than expected. Some of the major challenges related to the 2D nanopore devices are depicted in Supplementary Fig. 3a. Of all the unsuccessful devices (*n* = 36), ~70% of the nanopore devices showed unstable MoS_2_ nanopore as a major reason for the device failure. This issue has also been observed in graphene nanopores^6^, where ~30% of the device failure is attributed to membrane damage. Indeed, 2D membrane and nanopore stability becomes of prime importance for the practical applications of the 2D nanopore sensors. Other issues include improper nanopore wetting, that refers to the first nanopore device which is outright difficult to wet, and such a device remains unwet for a longer period of time despite alcohol pre-wetting or electrowetting. On the other hand, ‘clogging’ refers to those devices that produced linear I–V characteristics but clogged permanently during the course of measurement. The reason for such clogging is often due to a nanobubble and/or polymer or hydrocarbon-related contamination leading to device failure.

Figure 1g–h shows a schematic representation of two prime reasons for instability in 2D nanopores: (1) defects or leaky unstable membrane forming cracks and delamination, and (2) oxidative dissolution of a 2D nanopore in an air-saturated aqueous ionic solution.

### Ionic measurements and delamination of monolayer MoS_2_

**Fig. 2:**
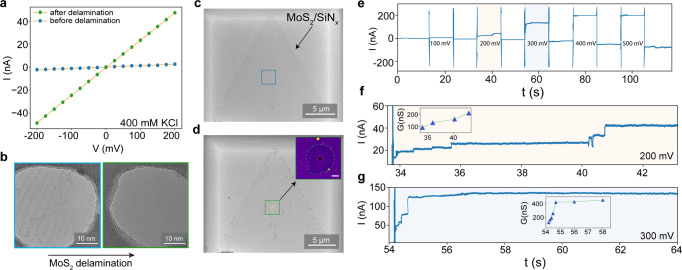
Delamination of monolayer MoS_2_ from the SiN_x_ surface. **a** An I–V curve from Device 1 measured in 400 mM KCl (pH 8) from MoS_2_ nanopore with ~13 nS in the beginning that increases to ~225 nS (conductance corresponds to the size of the SiN_x_ aperture). **b** Bright-field TEM images of Device 1, before and after the delamination. **c**, **d** A TEM image with a large field of view of Device 1 shows local delamination around the aperture area. Inset in (**d**) is a false-color zoom-in image with an area where the MoS_2_ is completely detached (depicted as a dotted area) while the surrounding area retains MoS_2_. Scale bar, 200 nm. **e** Measurements on Device 2 (d_TEM_ ~2.5 nm). Experimental ionic traces show an unstable MoS_2_ pore current probed at different voltages (range: +/−500 mV, measured every 100 mV for 10 s). **f**, **g** Zoomed-in traces show an abrupt increase in the ionic current at low voltages: 200 mV and 300 mV. Insets in respective figures show a stepwise increase in the current which is voltage-dependent.

To extract nanopore sizes from ionic current we used the general conductance model^40^. Figure 2a shows an I–V response of a small nanopore in the MoS_2_ monolayer membrane. The device initially showed pore conductance (G_open_) as ~13 nS (bulk conductivity of the solution = 4.12 S/m) which corresponds to the calculated nanopore diameter of ~4.2 nm considering membrane thickness (L = 1 nm). After a few minutes of measurements, we observed an unstable ionic trace, and the G_open_ shoots up to ~225 nS (d_calc_ = ~72 nm, L = 25 nm). The d_calc_ corresponds to the open pore current of a bare aperture from the SiN_x_ membrane. For comparison, we measured the leakage conductance of the intact SiN_x_ membrane to be lower than ~300 pS (Supplementary Fig. 4). Indeed, bright-field TEM analysis of the same device reveals that the monolayer MoS_2_ membrane got detached or delaminated from the aperture (Fig. 2b). Figure 2c, d shows a large field of view TEM image of the same device with MoS_2_ on the membrane before and after delamination near the aperture, respectively. The TEM image of MoS_2_ delaminated from the aperture area on the membrane suggests weak interaction of MoS_2_ to the underlying SiN_x_ surface (Fig. 2d green square and inset). A similar abrupt increase in the open pore ionic current was also observed with Device 2 which has a single MoS_2_ nanopore of ~2.5 nm fabricated by TEM drilling (Supplementary Fig. 5). Figure 2e shows experimental ionic traces probed up to 500 mV measured in 1 M KCl. The ionic current trace follows a similar pattern as Device 1, the current increases in a stepwise manner starting at 200 mV and more. Figure 2f, g show zoomed current traces from two voltages: 200 mV and 300 mV where the current increases in discrete steps. We also observe the stepwise increase in the pore current up to G_open_ ~150 nS (200 mV) and even up to 400 nS (at 300 mV). This conductance is higher than the expected G_open_ from this device which is around ~25 nS (L = 1 nm, bulk conductivity of the solution = 11.5 S/m). Hence, pore instability can also occur within few minutes of measurements. We also observed unstable open pore ionic current and stepwise increase with voltage in different ionic strengths of the solution as shown in Supplementary Fig. 6.

Unstable ionic current trace or increase in ionic current several orders of magnitude more than expected can be attributed to one or more of the following reasons: the nanopore enlargement in size, or multiple nanopores formation at different defective sites in the 2D material^12,41^, or delamination of 2D material^9,36^. An abrupt increase in open-pore current has been observed before in 2D nanopores at higher voltages (>700 mV)^9,36,41^. As shown in Fig. 2e, f, the open-pore current at 300 mV shows more prominent increment steps compared to 200 mV indicating the delamination process is voltage-dependent. This corroborates well with studies on graphene pores transferred on hydrophilic SiN_x_ surfaces where delamination can get initiated at a voltage of ~250 mV, and the extent of delamination is voltage-dependent^36^. Supplementary Fig. 7 shows examples from three different MoS_2_ nanopore devices where a detachment of the monolayer was confirmed with TEM imaging. The 2D membrane instability via delamination can be influenced by an applied voltage and the adhesion strength between MoS_2_ and SiN_x_ surfaces.

### Substrate modification and enhanced 2D membrane stability

**Fig. 3:**
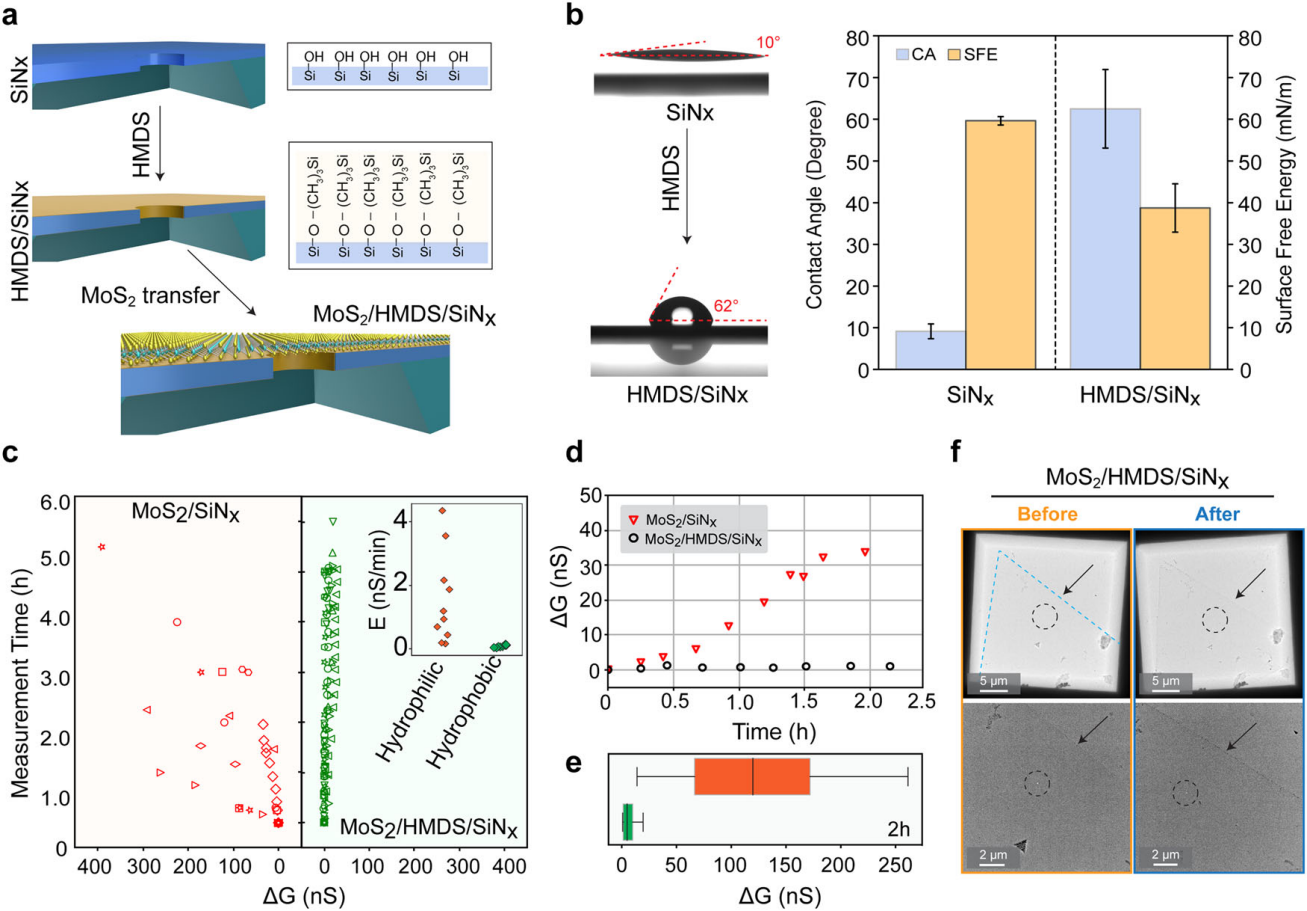
Enhanced extrinsic stability of MoS_2_ membranes using HMDS-modified SiN_x_ substrates. **a** Schematic showing stepwise coating of the SiN_x_ surface with HMDS followed by transfer of monolayer MoS_2_ over the membrane. **b** Characterization of HMDS-modified SiN_x_ substrates after HMDS-coating. The surface shows an increase in the contact angle (from ~10° to ~62°, *n* = 15) and a decrease in the free surface energy post-treatment (*n* = 11). **c** Stability analysis of MoS_2_ nanopores transferred on hydrophilic substrates (*n* = 9) and hydrophobic substrates (*n* = 10). The increase in the open pore conductance (△G) is measured over time to indirectly correlate with the nanopore stability. Each marker represents △G from individual nanopore devices. Inset shows the rate of pore enlargement between all the pores. **d** A representative example of such two devices shows a drastic increase in conductance compared to the HMDS-modified substrate. **e** Box-plot showing a wide distribution of △G from unmodified hydrophilic substrates compared to a narrow distribution of modified substrates. **f** Bright-field TEM images of monolayer MoS_2_ transferred on HMDS/SiN_x_ substrate show an intact membrane and no delamination.

One way to increase the membrane stability of the MoS_2_ layer on the SiN_x_ substrate is to reinforce the adhesion to the underlying substrate. To achieve this, we uniformly coat the SiN_x_ surface with HMDS and transfer monolayer MoS_2_ to form MoS_2_/HMDS/SiN_x_ substrates (Fig. 3a). We start with evaluating the effectiveness of HMDS treatment by assessing the change in wettability of the SiN_x_ surface. As shown in Fig. 3b, we calculate the contact angle (CA) and extract surface free energy (SFE) of the HMDS/SiN_x_ surface using the Extended Fowkes method^42^.

Surface hydrophilicity is achieved through piranha solution treatment which is generally used to clean the nanopore devices. This treatment results in a formation of a dense and thin monolayer of hydroxyl groups (-OH) on the SiN_x_ surface^9,33^. Additionally, the SiN_x_ surface is also exposed to oxygen-plasma, which renders the surface hydrophilic, with CA, ~10° (Fig. 3b) while pristine SiN_x_ surface without any such treatment is ~48° (Supplementary Fig. 8). After HMDS treatment, the contact angle increases to ~60°, due to the exposed methyl groups (-CH_3_) being relatively more hydrophobic (Fig. 3b). As shown in Fig. 3b (right side), the SFE measurements show that HMDS-primed SiN_x_ surfaces (~40 mN/m) have lower surface free energy compared to the uncoated SiN_x_ surface (~60 mN/m), verifying successful HMDS-coating on the SiN_x_ surface. The HMDS-coating stability on the SiN_x_ surface in long-term storage for up to 28 days is shown in Supplementary Fig. 9.

We then set out to study the MoS_2_ nanopore stability by measuring the G_open_ for all the devices over time. As shown in Fig. 3c, we compare the change in the G_open_ (△G) from different MoS_2_ nanopores devices transferred on the conventional hydrophilic SiN_x_ substrates with HMDS/SiN_x_ substrates. A general membrane stability improvement is observed for the HMDS-modified MoS_2_ nanopore devices with low △G ( < 50 nS) compared to the unmodified devices where the △G increases more than 400 nS after 5 h of measurements. The inset shows a huge spread in the rate of change (E) in G_open_ in unmodified SiN_x_ devices (up to 4 nS min^−1^) compared to narrow distribution (<1 nS min^−1^). Figure 3d, shows two MoS_2_ nanopore devices with △G increasing for MoS_2_/SiN_x_ versus MoS_2_/HMDS/SiN_x_ surface for about 2 h of measurements. It is obvious that within the same measuring time interval, the conductance of the unmodified device increases to around 40 nS, while that of the HMDS-modified device maintains stable conductance value. Figure 3e shows a two-hour experiment variation in the distribution of △G in 1 M KCl for all the measured devices. Supplementary Fig. 10 shows examples of I–V characteristics of five MoS_2_ nanopore devices coated with HMDS. Occasional decrease of conductance is possible because of nanopore clogging that is frequently observed for 2D nanopores, which can be caused by nanobubbles, hydrocarbons, and other impurities in the buffer solution^9^. In our experience with MoS_2_ nanopores, generally, such kind of clogging can be unclogged by applying a reverse polarity voltage bias^9^ or re-flushing with a degassed and filtered aqueous solution.

The improvement in the 2D membrane stability after a surface modification indicates that 2D material-substrate interaction is of critical importance. Due to the enhanced van der Waals force between the hydrophobic MoS_2_ layer and the HMDS-modified substrate, we observe a prolonged lifetime of the MoS_2_ film on nanopore devices. Figure 3f shows a TEM image of a device with an intact film of a monolayer MoS_2_, before and after the experiment. The cleanliness and image of the nanopore are shown in Supplementary Fig. 11. For the MoS_2_/HMDS/SiN_x_ device, the MoS_2_ layer was intact as shown with arrows on the same area (Fig. 3f). The MoS_2_/HMDS/SiN_x_ interaction-related stability performance emphasizes the detachment of MoS_2_ from the substrate is one of the major factors that causes device failure. Therefore surface modification strategies like HMDS-coating reinforces 2D layer interaction with the substrate and high membrane stability.

### Oxidation of MoS_2_ and nanopore expansion in aqueous solution

The aging of atomically thin materials due to oxidation is a major challenge in the field of 2D layered materials^34,35^. Oxidation degrades the electronic and chemical properties of 2D TMDs and limits their application. It has been observed that in ambient conditions, the oxidation process of MoS_2_ can start from the defects, edge planes, and grain boundaries resulting in the etching of the monolayer^34^. The oxidation process can occur by a thermodynamically more favorable reaction where the O-atom first adsorbs onto a S-atom from the basal plane of the MoS_2_ followed by a substitution reaction to form a Mo-O bond^38^. In comparison to the so-called ‘air-sensitive’ 2D materials^37^, monolayer MoS_2_ is generally considered to be relatively stable as the basal plane faces a high energy barrier for oxygen molecules to diffuse in ambient conditions^38^. The high energy barrier (~1.59 eV) protects the basal plane from molecular adsorption and substitution of S-atoms by O-atoms in pristine MoS_2_. However, the barrier decreases to ~0.8 eV in the presence of reactive sites such as vacancies or other defects^38^. Since the initial number of defects in the pristine MoS_2_ can influence the rate of oxidation and degradation, we first set out to quantify pristine defects in our samples. We study 2D material quality both qualitatively and quantitatively in terms of the number of defects in the pristine monolayer MoS_2_ (both monocrystalline or large-area grown MoS_2_) used throughout the study.

**Fig. 4:**
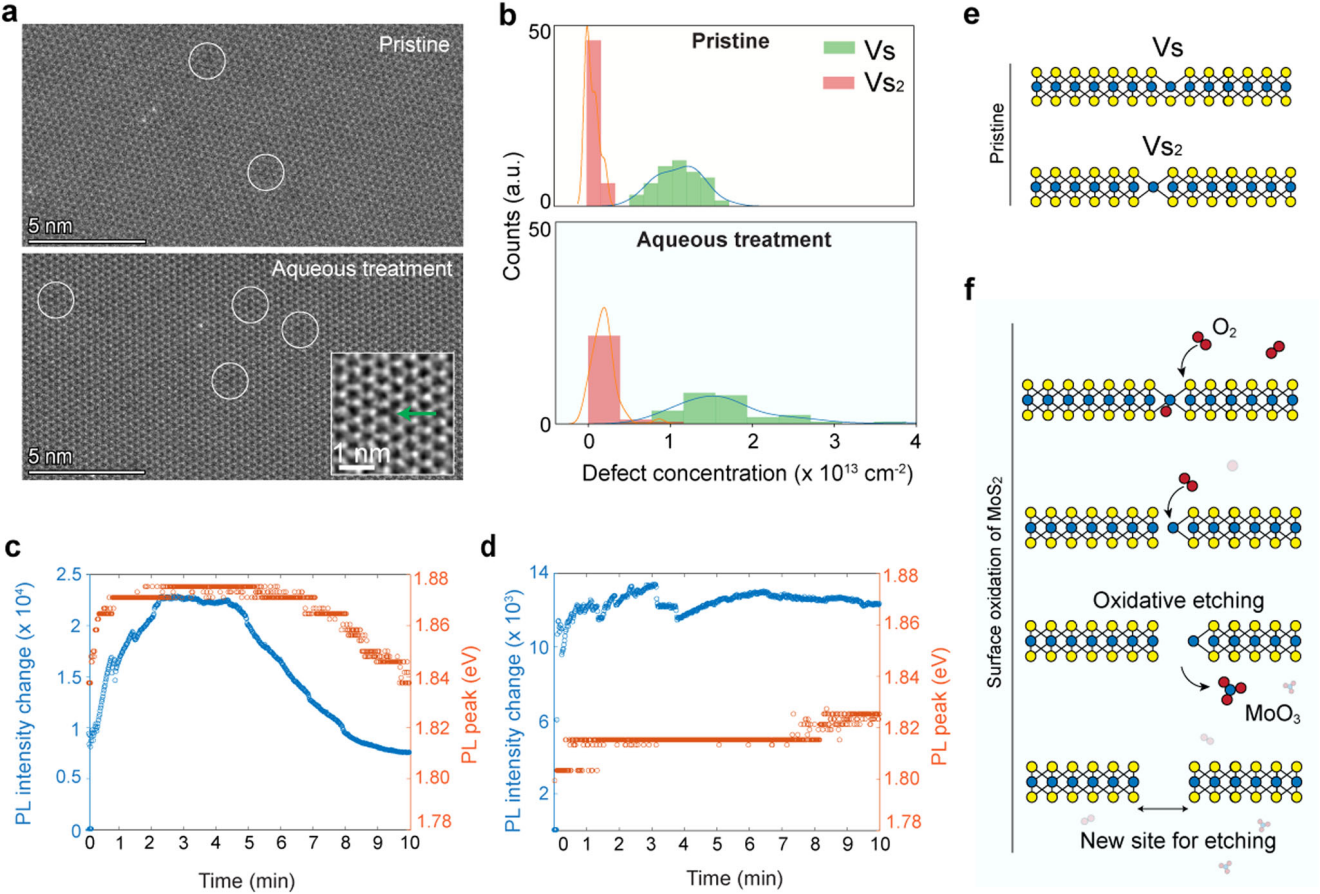
Quantification of surface defects and oxidation of monolayer MoS_2_ in pristine and aqueous solution. **a** Aberration-corrected ADF-STEM image of monolayer MoS_2_ in its pristine form and after incubation in aqueous solution. Marked circles show single sulfur vacancies in MoS_2_ (Vs). Inset, an example of the marked circle, showing a single sulfur vacancy defect (green arrow). **b** Histogram and kernel density estimation analysis show two primary defect populations (single sulfur vacancies annotated as Vs and double sulfur vacancies, Vs_2_). There is a slight increase in the sulfur defect concentration after treatment on the same order of magnitude at the same imaging conditions. PL spectrum of MoS_2_ in aqueous solution the presence of dissolved oxygen (~8 mg L^−1^) shown in **c**, and reduced oxygen level (~1 mg L^−1^) as shown in **d**. **e** Chemical structure of pristine MoS_2_ showing sulfur vacancies in the basal plane. **f** Schematic showing oxidative dissolution and etching of monolayer MoS_2_ in air-saturated aqueous solution.

Figure 4a shows the quantification of the defects of MoS_2_ used for nanopore experiments. Detailed analysis of initial defect density calculation and quantification of defects in MOCVD large-area MoS_2_^33^ is shown in Supplementary Fig. 12 and Supplementary Fig. 13, and in the Materials and methods section. We compare the sulfur defect concentration in pristine MoS_2_ with the new defects introduced by incubation in an aqueous ionic solution (non-degassed 1 M KCl, ~12 h). Aberration-corrected TEM (Fig. 4a, left panel) shows a representative TEM image of the same MoS_2_ sample before and after an aqueous treatment (12 h). The total sulfur defect vacancies (Vs + Vs_2_) is estimated from ~3500 nm^2^ suspended area of monolayer MoS_2_ either in pristine form or post-incubation in an aqueous solution. The sulfur defect concentration increased from 1.2 ± 0.3 × 10^13^ defects cm^−2^ to 1.9 ± 0.4 × 10^13^ defects cm^−2^ after incubation in an aqueous solution with dissolved O_2_ level (8 mg L^−1^) (Fig. 4b).

The dissolved O_2_ in water thus plays an important role in inducing defect formation (~0.7 × 10^13^ defects cm^−2^ in 12 h) and could thereby influence the stability of the 2D MoS_2_ in an aqueous environment^34,35^. We study the accelerated oxidation process using photoluminescence spectroscopy (PL) on monolayer MoS_2_. The MOCVD-grown MoS_2_ was transferred on a clean glass substrate and the PL spectrum of MoS_2_ is recorded in an aqueous solution. Figure 4c shows the changes in the PL spectrum of MoS_2_ in water under laser excitation. After 4 min of laser illumination, the spectral peak intensity increases by more than two fold, and the photon energy blue-shifts by ~35 meV. Such a spectral shift corresponds to the transition from charged exciton emission to exciton emission that is caused by a reduction of free electrons in *n*-type MoS_2_^43^. We suspect that the dissolved O_2_ molecules in water (~8 mg L^−1^) react with MoS_2_ under laser illumination as oxygen is an electron-withdrawing species. After the initial 4 min, a decay of PL intensity and spectral red-shift of MoS_2_ is observed in the presence of dissolved oxygen (Fig. 4c).

A plausible cause could be a local material dissolution as similar spectral behaviors and mechanisms have been reported on MoS_2_ exposed to air^44,45^. To verify our hypothesis, we reduced the dissolved oxygen level in the water below 1 mg L^−1^ by argon gas purging and then performed the spectral measurement on MoS_2_ in a sealed chamber. As shown in Fig. 4d, the PL spectrum of MoS_2_ is stable in both intensity and energy throughout the measurement, implying neither photo-induced chemical reaction nor plausible material dissolution. This is in stark contrast with the spectral shift of MoS_2_ in the presence of dissolved oxygen (~8 mg L^−1^). Figure 4e, f shows a schematic representation of single and double sulfur vacancies, and oxidation-induced etching of monolayer MoS_2_, respectively.

**Fig. 5:**
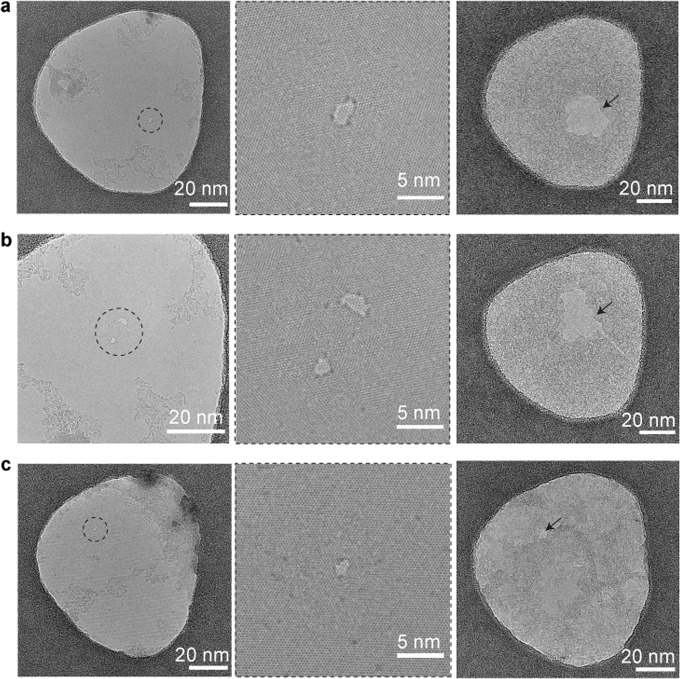
Bright-field TEM images of monolayer MoS_2_ nanopores (single pore or double pores) drilled using TEM. All the devices used here are MoS_2_/HMDS/SiN_x_ substrates. The pores were incubated in 1 M KCl aqueous buffer (10 mM TE-buffer, pH = ~7.5) for 12 h at room temperature (~20 °C). The dissolved O_2_ concentration was measured as ~8 mg L^−1^ in panels (**a**, **b**) and maintained at ~1 mg L^−1^^11^ in panel (**c**). The dotted circles show the pore area and arrows point towards the same enlarged pore area.

Further, we study the oxidation-related stability and the dissolution by reducing the amount of oxidizing agents in the aqueous buffer. As shown in Fig. 5, nanopores (single or double pores) in monolayer MoS_2_ devices on HMDS-coated substrates are fabricated in TEM, and the pore expansion is studied during incubation in an aqueous solution without applying any external voltage. As seen in Fig. 5a, b, the nanopores enlarged in size when incubated in an air-saturated non-degassed 1 M KCl TE-buffer (pH ~7.5) at ambient temperature (~20 °C) for 12 h. Whereas the single nanopore incubated at in low O_2_-concentration (~1 mg L^−1^) buffer showed a slight increase in pore size (Fig. 5c). More quantification of pore growth and TEM images are shown in Supplementary Fig. 14. The noise comparison of the devices used are shown in Fig. S15.

Previosuly, bulk layered MoS_2_ (~2 µm particles) has shown high stability to oxidation in an air-saturated aqueous solution^38,39,46^. While in 2D MoS_2_ monolayers are more prone to oxidative degradation in an aqueous solution, especially at the nanopore sites as seen in Fig. 5. Single MoS_2_ nanopore from the same device grew in the air-saturated buffer and the double nanopores grew and merged to form a single larger nanopore (Fig. 5a, b). The aqueous oxidation of MoS_2_ is typically caused by the presence of oxygen and hydroxyl ions in the aqueous solution that can etch MoS_2_ via dissolution products such as MoO_3_ and MoO_4_^2−^ ions^35,38,46,47^.

### Long term DNA sensing

With enhanced 2D membrane stability and by reducing aqueous oxidation of the monolayer MoS_2_, we then set out to measure the stability of the nanopore in combination with DNA sensing. Single-molecule measurements using a molecular ruler, such as DNA, can be used as a tool to study changes in nanopore conductance^8,10^. Under the influence of an electric field, negatively charged DNA can be driven toward the pore, and a successful passage through the pore generates a resistive pulse called an ‘event.’ Statistical measurements of conductance drop (G_drop_) of such events can indicate the membrane thickness as well as the size of the nanopore. Since for our study, we employ nanopore in a monolayer MoS_2_, by considering a constant thickness, such statistical analysis of events can help us to probe the changes in the nanopore size throughout the experiment. This analysis is particularly useful in cases where the size of the nanopore is comparable to the size of translocating molecule. The changes in G_drop_ over time can indicate if the nanopore got enlarged, or also new nanopores have been created.

We perform continuous monitoring of the nanopore size using DNA translocations in monolayer MoS_2_ nanopore fabricated using TEM drilling at 80 kV^9^. The TEM image of and the I–V characteristics of the nanopore are shown in Supplementary Fig. 16. The flowcell was completely sealed and the 1 kbp double-stranded DNA is translocated on the same pore for >3 h at 500 mV in low O_2_ concentration (<1 mg L^−1^) 1 M KCl TE buffer. The translocation events were analyzed using Open Nanopore (Python Package)^9^ and events were further fitted using the cumulative sums (CUSUM) algorithm^48^. Only the CUSUM-fitted events were further used for analysis and plotting that represent individual translocations of DNA molecules.

**Fig. 6:**
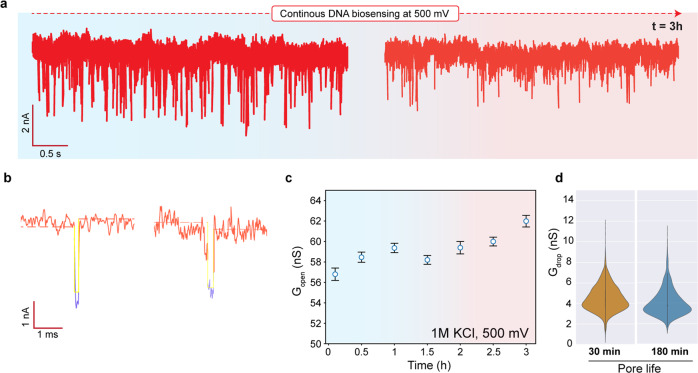
Long term DNA sensing using a monolayer MoS_2_ nanopore (*d* = 6.5 nm in 1 M KCl, pH 8) with HMDS-coated substrate. The flow cell is sealed during the experiment and the O_2_-concentration in the buffer is less than 1 mg L^−1^. **a** Translocation traces of 1kbp DNA at the beginning (number of events = 1832) and the end of the measurement (number of events = 1195) at 500 mV. **b** Example events from the traces in (**a**). The dotted line is a mean fit to open pore current and the yellow fit represents CUSUM-fit to the event. **c** Changes in the open pore conductance (G_open_) across different time points over the course of measurement. **d** Violin plots showing the distribution of conductance drop due to DNA molecules translocating through the pore at different time points. The conductance drop distribution shows a median value of 4.2 nS (interquartile range of 1.66 with upper adjacent value of 7.5 and lower adjacent value of 1.3) and 3.9 nS (interquartile range of 1.69 with upper adjacent value of 7.4 and lower adjacent value of 1.6) for 30 min and 180 min pore life, respectively.

In Fig. 6a, a typical raw trace of a double-stranded DNA (1 kbp) translocation events from a MoS_2_ nanopore of ~6.5 nm diameter is estimated from the open pore current. The calculated open-pore conductance (G_open_) at the beginning of the measurement was ~58 nS which increased to the G_open_ of ~62 nS towards the end of measurement at an expansion rate of 0.03 nS min^−1^. Since monolayer MoS_2_ was transferred for the experiment, we consider the thickness of the MoS_2_ monolayer membrane as L = 1 nm (including the hydrodynamic layer) for our analysis. Figure 6b shows examples of the individual translocation event from the respective traces. Figure 6c shows the mean G_open_ from the nanopore over the course of the analysis. We observe that the G_open_ of the nanopore grew by 4 nS (~7%) over 3 h of measurement. The conductance blockades for the DNA (2.2 nm) are then extracted from each of these events and represented as conductance drops (△G_drop_). The △G_drop_ was obtained from the same nanopore for traces at the beginning (*t* ~0–30 min) and the end (*t* ~150–180 min) of the measurement time. The translocation events (at least 10^3^ events) from these representative time frames are chosen to scale nanopore size based on the △G_drop_ obtained due to possible enlargement of the same nanopore.

As shown in the violin plots in Fig. 6d, the mean experimental value of the △G_drop_ from an unfolded DNA is △G_drop_ ~4.41 nS (number of events = 1832 events) and △G_drop_ of ~4.2 nS (number of events = 1195 events) at 30 min and 180 min respectively. These experimental values are closer to the expected △G_drop_ values of ~4.5 nS and ~4.3 nS, respectively for a membrane thickness (L = 1 nm)^40^. Supplementary Fig. 17a, b shows scatter plot and dwell time characteristics for events shown in Fig. 6d. We also observed unfolded, partially folded, and fully folded dsDNA configuration as shown as example events in Supplementary Fig. 17c. Such folded events have been observed before for dsDNA in 2D nanopores^7,10,13^, and more complex translocation conformations in SiN_x_ pores^49–51^. The △G_drop_ of folded dsDNA configuration with partially (or completely folded) configuration produced a △G_drop_ of ~7 nS (Supplementary Fig. 17c).

Previously, Larkin et al.^31^ demonstrated the stability of nanopores in thin HfO_2_ (2–7 nm) for continuous single-stranded DNA measurements. They also observed a G_open_ < 10% change in the conductance of a 1.4 nm diameter in HfO_2_ pore at 350 mV. Indeed, despite being only three atoms thin, we observe similar stability in monolayer MoS_2_ nanopore (~6.5 nm) at 500 mV enabling long term measurements. Long term stability also emphasizes the absence of an opening of additional pores on the free-standing area and good quality of our MOCVD-grown 2D material (fewer defects)^30^. Although the latter is highly dependent on the quality of the 2D material and experimental condition. Additionally, as discussed above, a stable open pore current highlights the strong interaction of monolayer MoS_2_ with HMDS-modified substrate.

We have studied major mechanisms of nanopore instability in 2D MoS_2_ nanopores and demonstrated methods to avert them. We propose a device fabrication protocol that enhances the stability of the monolayer MoS_2_ membranes in an ionic aqueous solution by introducing a layer of HMDS on the SiN_x_ surface, which improved the adhesion of MoS_2_ to the substrate. Further, we study the chemical oxidation in monolayer MoS_2_ using PL, and examine the 2D nanopore enlargement in ionic solutions. We demonstrate the nanopore growth can be minimized by reducing the oxygen level in the ionic buffer in standard nanopore experimental conditions. Finally, we show continuous DNA translocation measurements on the same pore for hours with high stability. The stability of atomically thin free-standing 2D nanopores in ionic solutions is currently a major hurdle in the development of 2D nanopore sensors. With proposed stabilization methods, 2D nanopores can be used as reusable sensors and pave the way toward high-throughput long term biosensors.

## Methods

### Wafer-scale substrate fabrication

Double-side polished 100 mm (orientation: <100>) undoped Si-wafers (Active Business) were covered with 60 nm of SiO_2_ and 20 nm low-stress SiN_x_ from both sides. Photolithography and dry etching were done to open apertures in the back side SiN_*x*_ layer for the following wet etching process required for SiN_*x*_ membrane formation on the front side. Front-side e-beam lithography (Raith EBPG5000 + ) and dry etching were performed to form 80 to 120 nm-diameter apertures in SiN*x* membranes with the following parameters: 100 keV e-beam, polymethyl methacrylate (PMMA, molecular weight 495 K, 4% in anisole) as an e-beam resist and CHF_3_/O_2_ gas mixture for dry etching. As a final step, acid piranha cleaning and 300 °C baking were applied to achieve a clean surface of the target nanopore substrate prepared for the transfer of MoS_2_.

### MoS_2_ growth and transfer

The triangular shape monolayer MoS_2_ crystal was grown via metal-organic chemical vapor deposition (MOCVD) in a 2-inch quartz tube furnace. The c-plane sapphire was used as the growth substrate and pre-annealed at 1000 °C for 2 h in the air to create atomically smooth step terraces^52^. In order to suppress nucleation and promote large-area crystal growth, sodium chloride (NaCl) solution was spin-coated on the substrate prior to the growth^53^, as well as the introduction of oxygen during the growth^54^. The two gas precursors, molybdenum hexacarbonyl (Mo(CO)_6_) and hydrogen sulfide (H_2_S), carried by Ar gas, were mixed in the furnace with a flow rate ratio of 1:6028. The reaction took place at 850 °C under subatmospheric pressure (850 mbar) and lasted for 30 min. After the growth, the Mo(CO)_6_ precursor was immediately closed, while the H_2_S was continuously supplied during the whole cooling process to prevent the sulfur vacancy formation. The large-area, continuous MoS_2_ films that were used for initial defect density calculation were synthesized using the MOCVD method described elsewhere^33,55^. Transfer of monolayer MoS_2_ was performed using the PMMA-assisted transfer method described before^9^.

### Surface modification and characterization

The surface of the SiN_x_ substrate was modified following oxygen plasma treatment (Tergeo Plasma Cleaner, PIE Scientific) and a standard Bis(trimethylsilyl)amine ([(CH_3_)_3_Si]_2_NH, HMDS) priming process (OPTIhot VB20 HMDS unit, ATMsse). The oxygen plasma treatment was done to improve the HMDS priming efficiency by introducing more hydroxyl groups (-OH) on the SiN_x_ surface, with the following parameters: 35 W RF Power, 50 mtorr vacuum state, with 5.0 sccm O_2_ gas flow for 20 s. The standard HMDS priming process started with 10 min dehydration at 135 °C in a vacuum chamber to remove the moisture. After dehydration bake, the surface was then exposed to the vapor HMDS for 60 s. A monolayer of HMDS will be deposited on the SiN_x_ surface after the -OH groups on the wafer surface reacted with amino groups (-NH) from HMDS, and the surface was therefore terminated with methyl groups (-CH_3_), which makes it hydrophobic^56^. After the HMDS vapor exposure, several pumping, and N_2_ purging cycles were followed to remove the residual HMDS atmosphere. After the process was complete, substrates were removed from the chamber and after cooling down to room temperature, they were stored in a vacuum before the transfer process.

The contact angles (CA) and surface free energies (SFE) were obtained through a multi-dosing and imaging system (DSA-30E, Krüss) before and after the HMDS surface modification process to demonstrate the effectiveness of the priming process. The measurements started by depositing a drop of liquid on the sample surface, and the computation of CA was done on the live image or a captured frame by sequentially determining the baseline, extracting the liquid profile, and then calculating the angle. Three different liquids were used for the measurements with recommended doses, including water (3 μL), diiodomethane (2 μL), and ethylene glycol (2.5 μL). The CA values usually refer to measurement results from only water. SFE was also calculated on the system based on the CA values of three kinds of liquid using the Extended Fowkes method^42^.

### TEM characterization and quantification of defects

Aberration-corrected annular dark-field scanning transmission electron microscopy (ADF-STEM) imaging was performed using a double Cs corrected FEI Titan Themis TEM 60–300 kV, equipped with Schottky X-FEG electron source and a Wein-type monochromator. All STEM were acquired using 21.2 mrad probe convergence angle, 185 mm camera length with corresponding 49.5–198 mrad collection angle, beam current of ~18–20 pA, and 8 µs dwell time with 512 × 512 pixels for the faster scans. For the image series, all the images were aligned using Image J. Intrinsic S defect concentrations were extracted from the linear fit extrapolation from defect concentration with respect to the accumulated e-beam dose rate^57^. To calculate the S-defect concentrations, different pristine regions were imaged (~3500 nm^2^ area) and defects were calculated manually.

### PL characterization

The PL spectrum of MoS_2_ in water was measured on a custom-built confocal microscope. Briefly, the monolayer MoS_2_ flakes grown by MOCVD in the batch as used in nanopore experiments were transferred on a coverslip^9^. The coverslip was then mounted on an air-tight fluidic chamber filled with Ultrapure MilliQ water with or without Ar gas purging. The fluidic chamber was then placed on top of the confocal microscope. A 561 nm laser (PicoQuant LDH-560) was focused on the MoS_2_ surface through a water-immersion lens (Olympus CFI Plan Apo, IR 60xc WI) with a power density of 3 × 10^5^ W/cm^2^. The spectrum of MoS_2_ was then measured by a fiber-coupled spectrometer (QE Pro from Ocean Optics). The dissolved oxygen level in water was measured in the fluidic chamber before and after spectrum measurement by a dissolved oxygen meter (Mettler Toledo InLab® OptiOx, part no. 51344621). All oxygen measurements were performed at ambient temperature (~20 °C).

### DNA translocation and analysis

The MoS_2_ nanopore chip was assembled onto a customized PMMA flowcell and details of which can be found here^9^. For pore size measurement and DNA translocations are performed in degassed and filtered 1 M KCl in TE buffer (pH ~8). Blank ionic traces were measured before checking artifacts or contaminants in the flowcell or from the substrate and the nanopore size using the conductance model^40^. We then add NoLimits 1 kbp DNA Fragment (50 nM, Thermo Fisher Scientific, USA) to the cis-compartment, and the flowcell is sealed. The DNA translocations are then recorded at a bias voltage of 500 mV. We exchange with fresh degassed buffer to avoid any salt evaporation effects on the open pore current. The oxygen concentration of the buffer was always monitored using a dissolved oxygen meter and reduced to less than 1 mg L^−1^. The event detection and fitting were performed using the Python-based OpenNanopore^9^ (https://www.epfl.ch/labs/lben/opennanopore-python).

### Supplementary information

Supplementary Material

## Data Availability

The data that support the findings of this study are available from the corresponding authors upon request.
