# Supplementary material for: High durability and stability of 2D nanofluidic devices for long-term single-molecule sensing
Source: NPJ 2D Mater Appl. 2023 Feb 23;7(1):11. doi: 10.1038/s41699-023-00373-5 (PMC11041726; doi:10.1038/s41699-023-00373-5)
Supplement: Supplementary file 1 — Supplementary Material [file 41699_2023_373_MOESM1_ESM.pdf]

Supplementary Information

High Durability and Stability of 2D Nanofluidic Devices for Long-term Single-Molecule Sensing

Mukeshchand Thakur,[1,*] Nianduo Cai,[1] Miao Zhang,[1] Yunfei Teng,[1,2,3] Andrey Chernev,[1] Mukesh Tripathi,[4] Yanfei Zhao,[4] Michal Macha,[1] Farida Elharouni,[1] Martina Lihter,[1] Liping Wen,[2,3] Andras Kis,[4] and Aleksandra Radenovic[1,*]

[1]Laboratory of Nanoscale Biology, Institute of Bioengineering, School of Engineering, EPFL, 1015, Lausanne, Switzerland

[2]CAS Key Laboratory of Bio-inspired Materials and Interfacial Science, Technical Institute of Physics and Chemistry, Chinese Academy of Sciences, 100190, Beijing, China

[3]School of Future Technology, University of Chinese Academy of Sciences, 100049, Beijing, China

[4]Laboratory of Nanoscale Electronics and Structure, Institute of Electrical Engineering and Institute of Materials Science and Engineering, School of Engineering, EPFL, 1015, Lausanne, Switzerland

*Corresponding authors: mukeshchand.thakur@epfl.ch, aleksandra.radenovic@epfl.ch

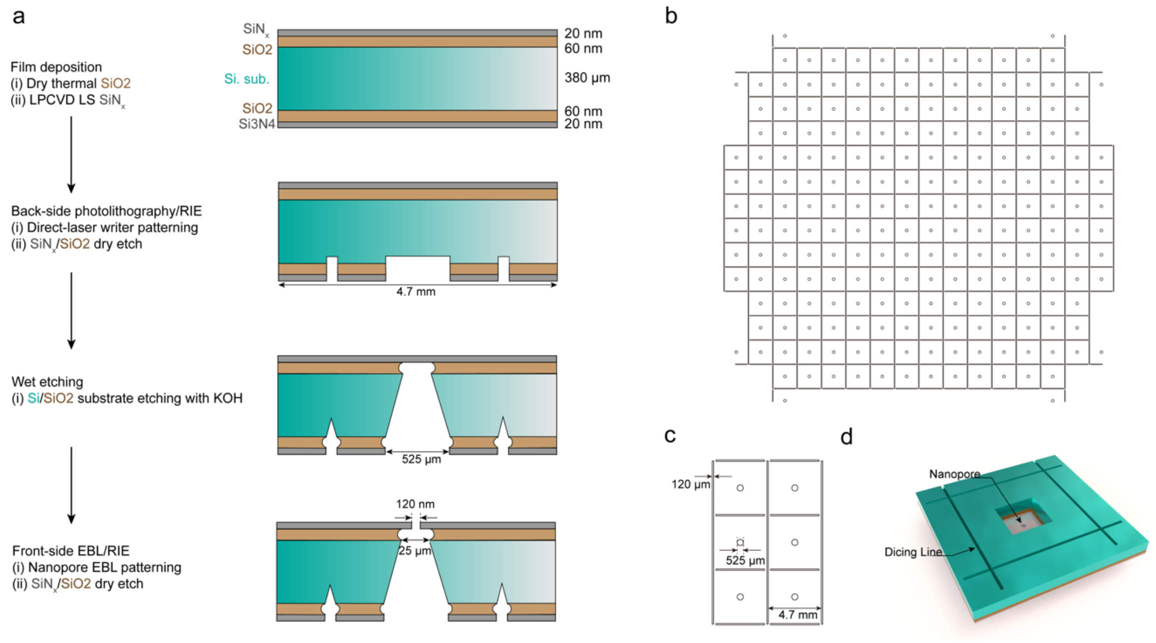

Supplementary Figure 1. The fabrication process flow of a 4-inch wafer-scale SiN_x nanopore chips fabrication. (a) Detailed fabrication process flow of SiN_x solid-state nanopore. (b) The pattern of a 4-inch wafer includes apertures for the SiN_x membrane and dicing line. (c) The detailed design of the wafer backside. (d) The schematic image of the solid-state nanopore from the backside.

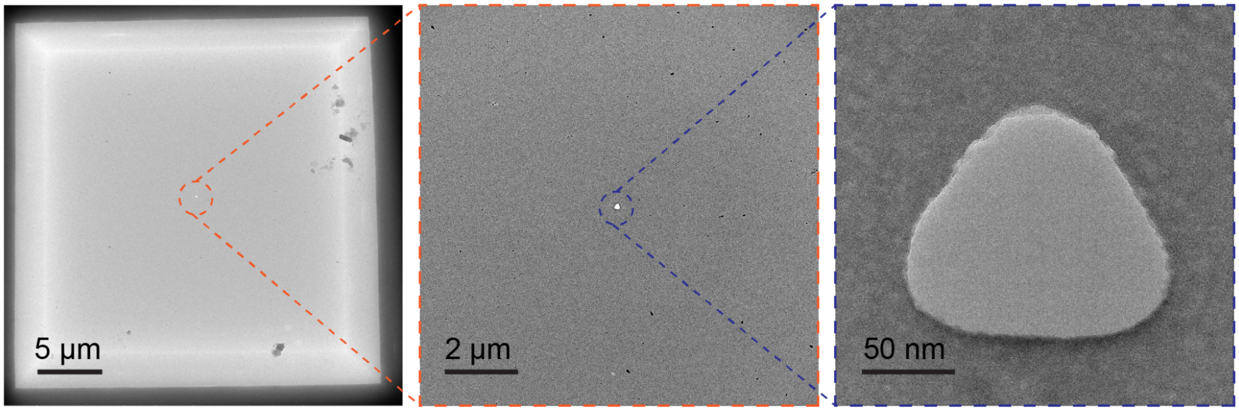

Supplementary Figure 2. Bright-field TEM image of SiN_x membrane with an aperture (dotted circles) defined by e-beam lithography.[1]

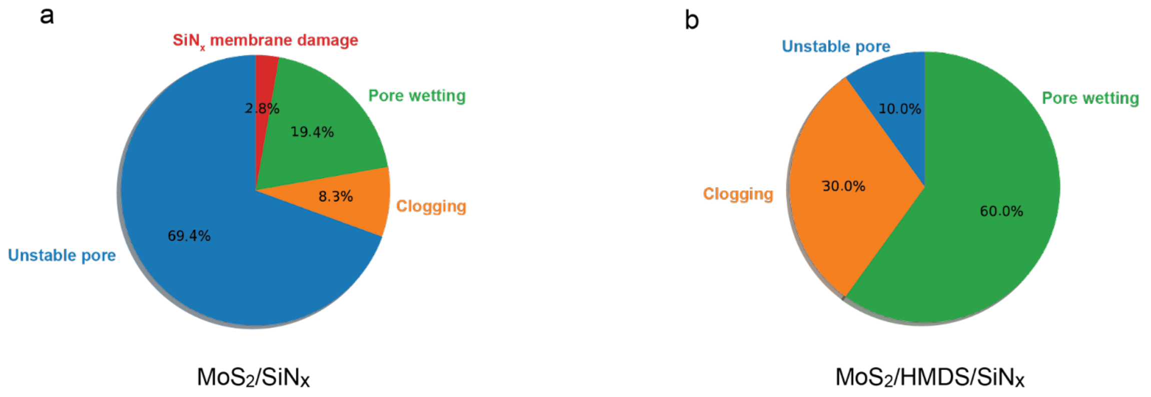

Supplementary Figure 3. Challenges related to the MoS_2 nanopore devices. For uncoated (MoS2/SiNx), a total 42 devices were measured, and 8 devices were successful while 36 devices were unsuccessful (device yield: 14.3%). The different reasons for the unsuccessful devices are shown in (a) dominated by an unstable pore. Whereas for HMDS-coated substrates (b), a total of 25 devices were measured of which 15 devices were successful and 10 devices failed due to reasons illustrated in (b). We observed that pore wetting (n=6) and clogging (n=3) were major reasons for the unsuccessful devices. Unsuccessful devices due to unstable pore however was reduced in aqueous ionic solution with a device yield of 56% (n=25) for MoS_2/HMDS/SiN_x devices.

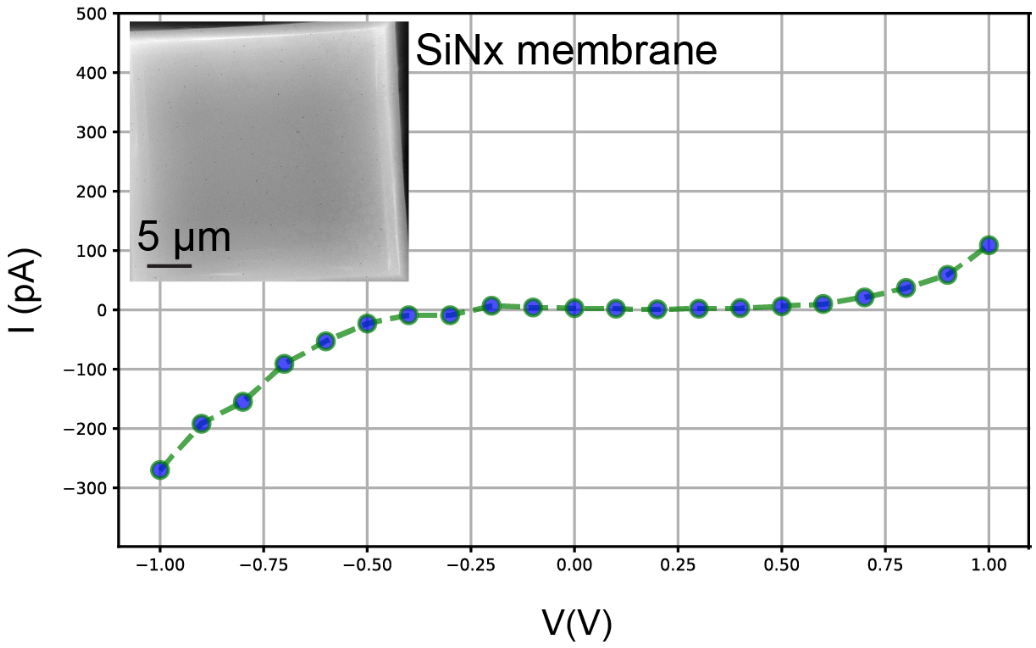

Supplementary Figure 4. I-V characteristic of a SiN_x membrane (20 nm thin) in 1M KCl. The bare SiN_x membrane shows a low leakage of <300 pS at an applied bias voltage of 1V. The current was measured after 50s for each applied voltage (-1 V to 1 V) to reduce the capacitive charging effects of the membrane.

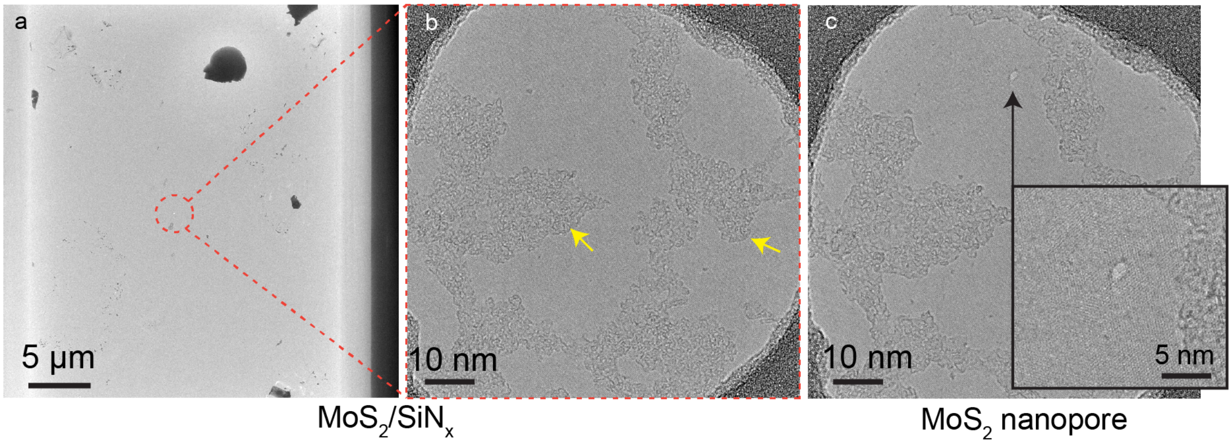

Supplementary Figure 5. Bright-field TEM image of Device 2 (Fig. 2e in Main Text), imaged and drilled at 80 kV. (a) Monolayer MoS_2 is transferred on the SiN_x membrane. The aperture is marked with a dotted circle and a high-resolution image of free-standing MoS_2 is shown in (b). The residues are hydrocarbons and PMMA polymer residues originating from MoS_2 transfer (shown by yellow arrows). (c) A single nanopore is created using a focused e-beam drilled on a clean part of the membrane. Inset, zoomed image of the single nanopore (~2.5 nm).

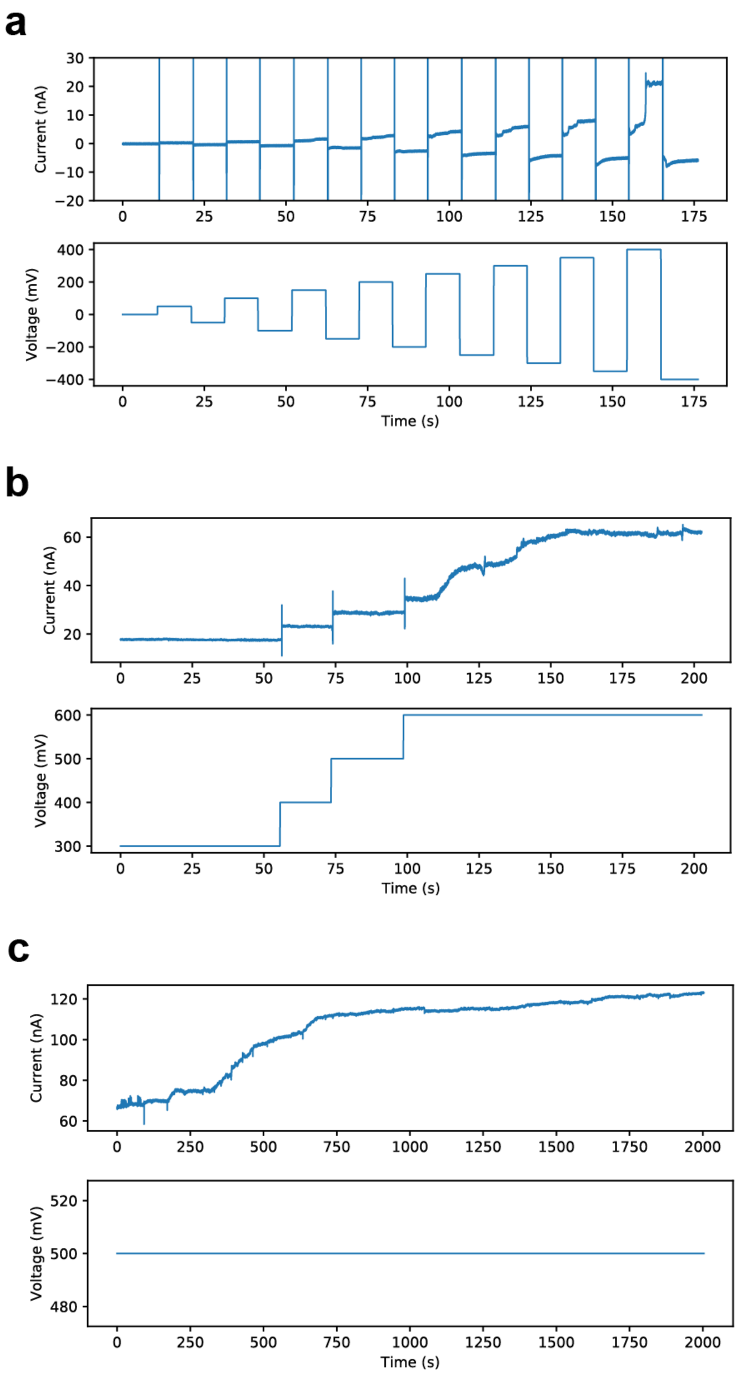

Supplementary Figure 6. Examples of unstable ionic current from MoS_2 nanopore devices. The ionic current traces were measured in (a) 400 mM KCl (Device 1, in the Main Text), (b) 1M KCl, and (c) 3M KCl in Tris-EDTA buffer (pH 7).

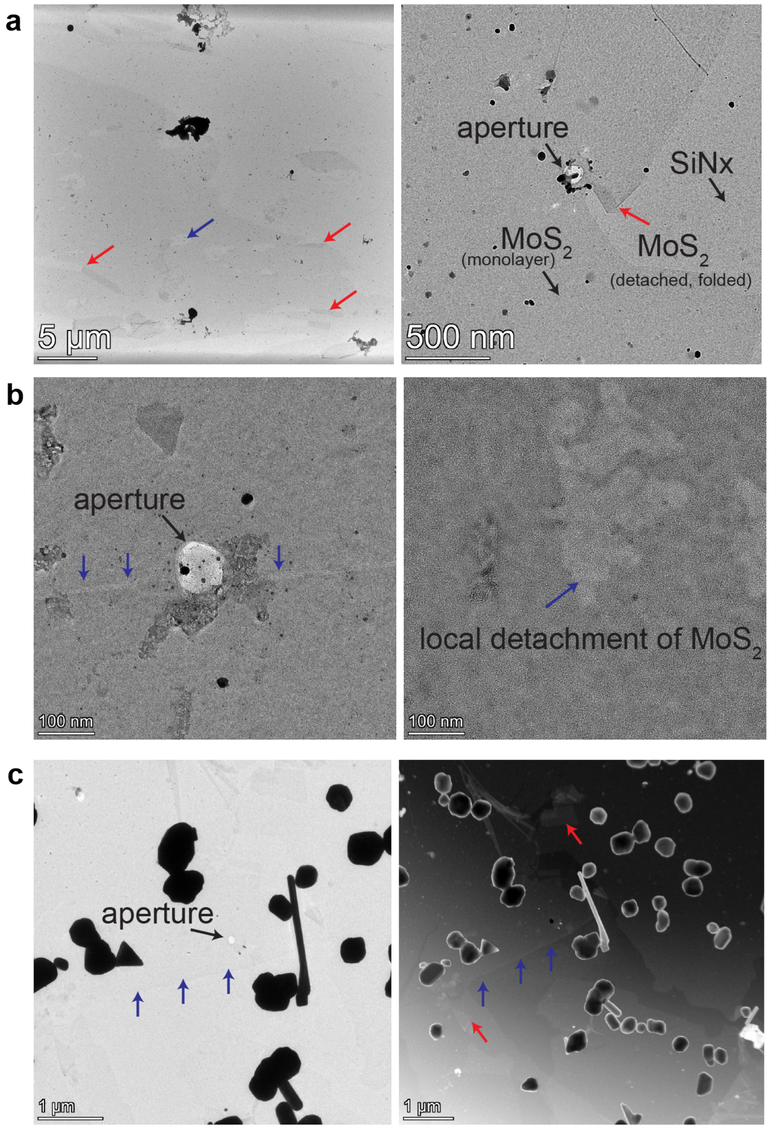

Supplementary Figure 7. TEM images showing local detachment of monolayer MoS_2 post-experiment in ionic aqueous solution from three different devices (a-c). The images were taken in bright-field TEM mode while STEM mode helps identifying the detached monolayers in some cases as shown in second panel in (c). The SiN_x aperture is shown with black arrows, detachment/cracks with blue arrows, and folding of the monolayer is shown with red arrows.

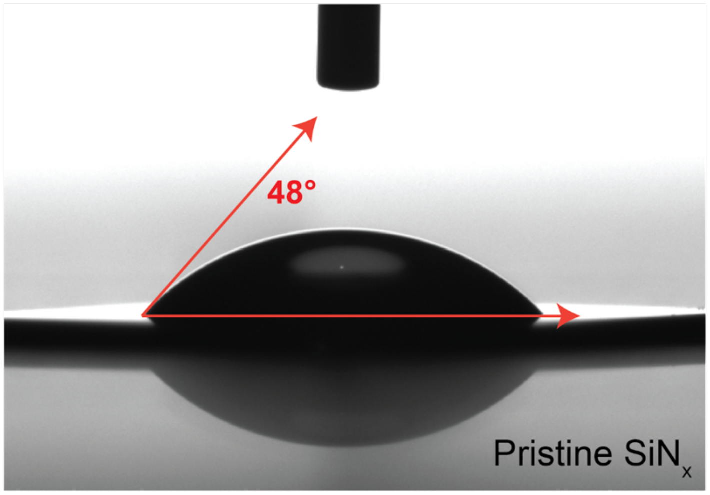

Supplementary Figure 8. An optical image showing contact angle of water on a pristine SiN_x wafer surface without piranha (or plasma treatment). The measured contact angle is 48.2 ± 3.5 degree, and the calculated surface free energy is 44.4 ± 1.0 mN/m (n=3).

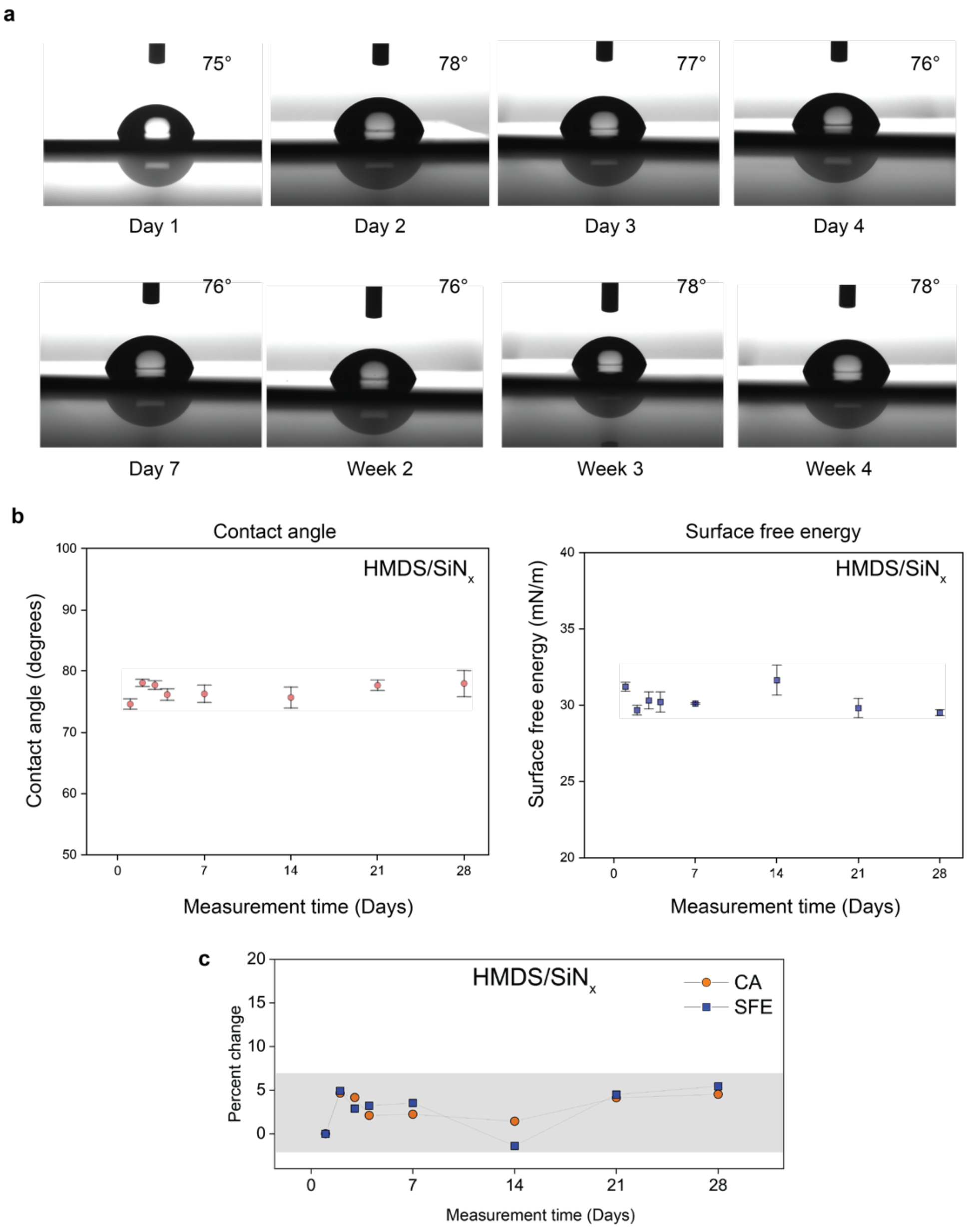

Supplementary Figure 9. Long-term HMDS-coating stability on SiN_x surface. (a) Contact angle measurement from the substrates over a period of 4 weeks. (b) Changes in the contact angle and surface free energy over time. (c) The HMDS coating remained stable with <7% change over a period of 28 days.

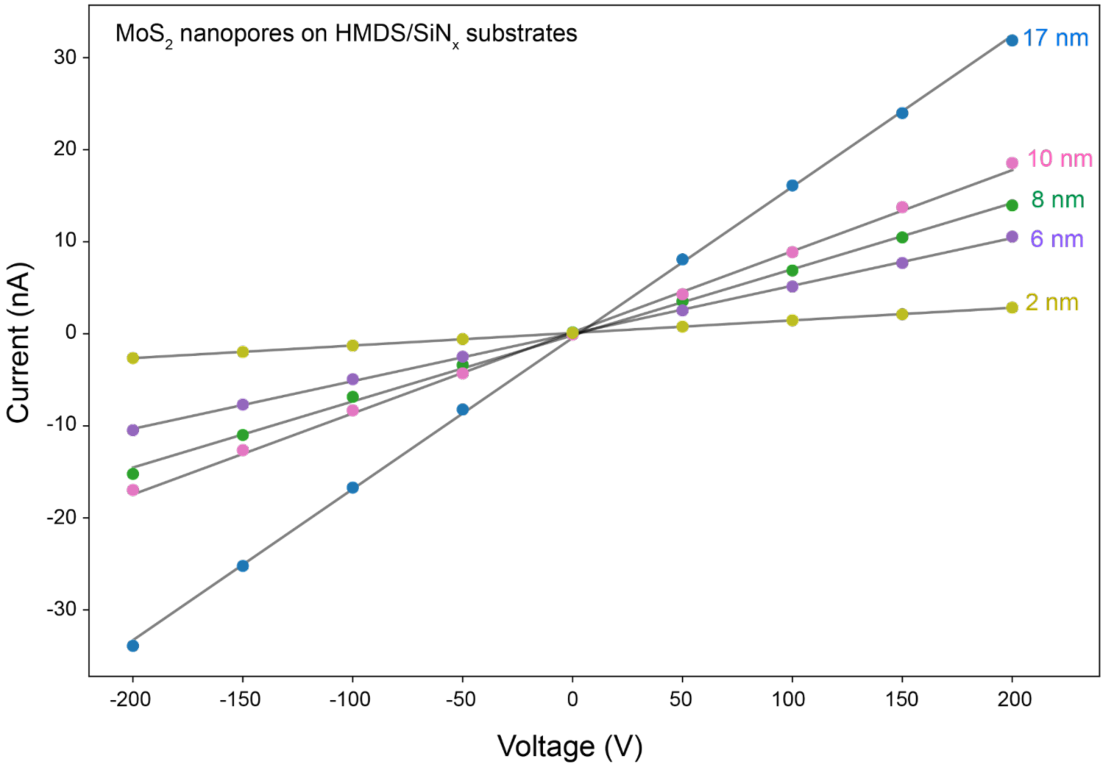

Supplementary Figure 10. I-V characteristics from five MoS_2 nanopore devices on HMDS coated SiN_x substrates. The pores correspond to different size ranges labeled in the inset.

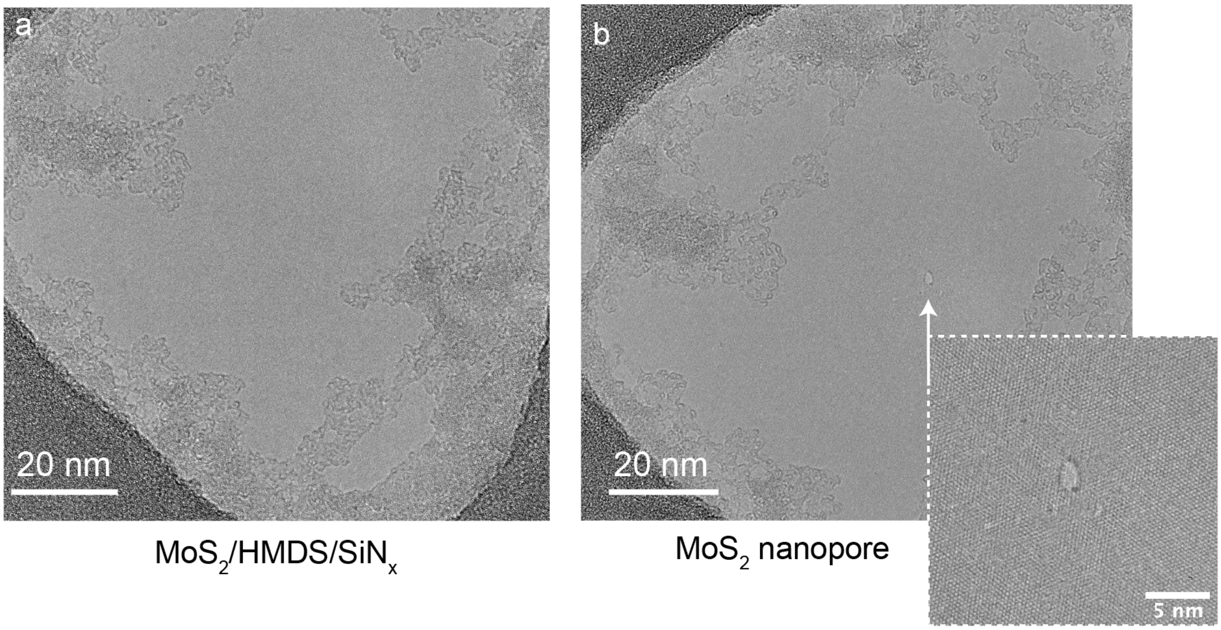

Supplementary Figure 11. Bright-field TEM image of MoS_2/HMDS/SiN_x from Fig. 3f in the Main text. (a) TEM image of the free-standing MoS_2 membrane after transfer. The device level of cleanliness was comparable to devices without HMDS treatment. Also, during imaging we have not observed any e-beam induced deposition that typically occurs due to contaminant. (b) After drilling a nanopore in monolayer MoS_2. Inset, zoomed image of ~3 nm MoS_2 nanopore.

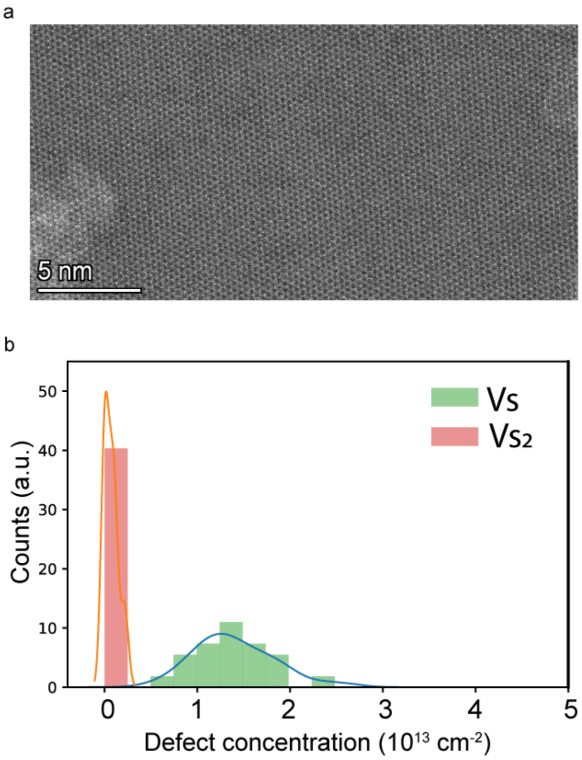

Supplementary Figure 12. Inherent S defect density calculations. (a) Aberration-corrected ADF-STEM image of monolayer MOCVD grown large-area MoS_2 layer. (b) Histogram and kernel density estimation analysis shows the distribution of Vs and Vs_2 defects.

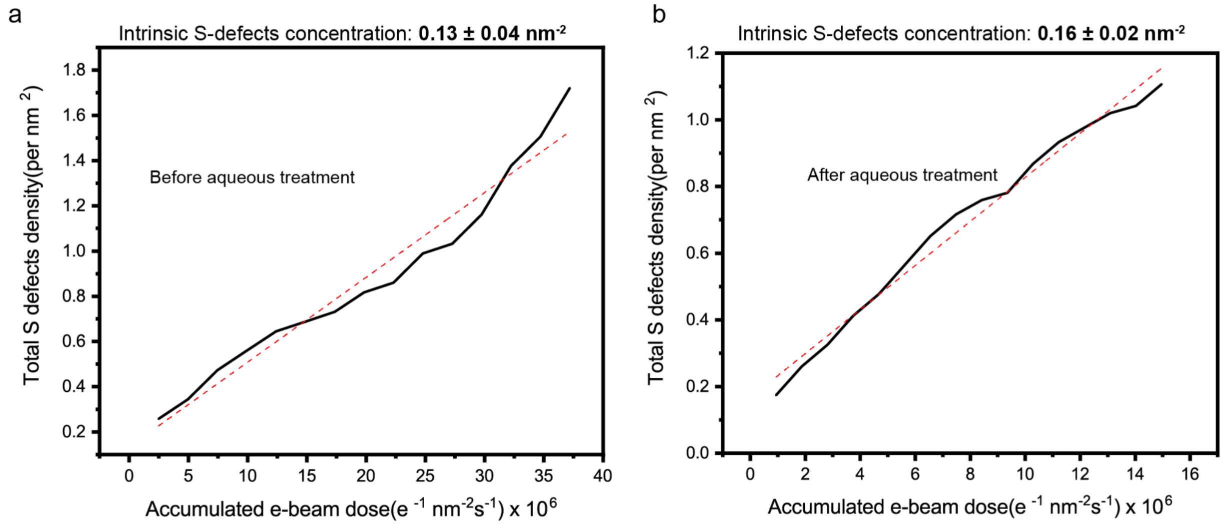

Supplementary Figure 13. Initial S-defect density calculation for the monolayer MoS_2 sample in Figure 4 in the main text. The measured defect densities (due to beam damage) are shown in black lines for different accumulated doses from an image series. A linear fit (dotted line) is used to extrapolate the total number of intrinsic S defects in the MoS_2 before the imaging. The total sulfur defects estimated are ~1.3 x 10^{13} defects cm^{-2} **(a)** and ~1.6 x 10^{13} defects cm^{-2} **(b)**.

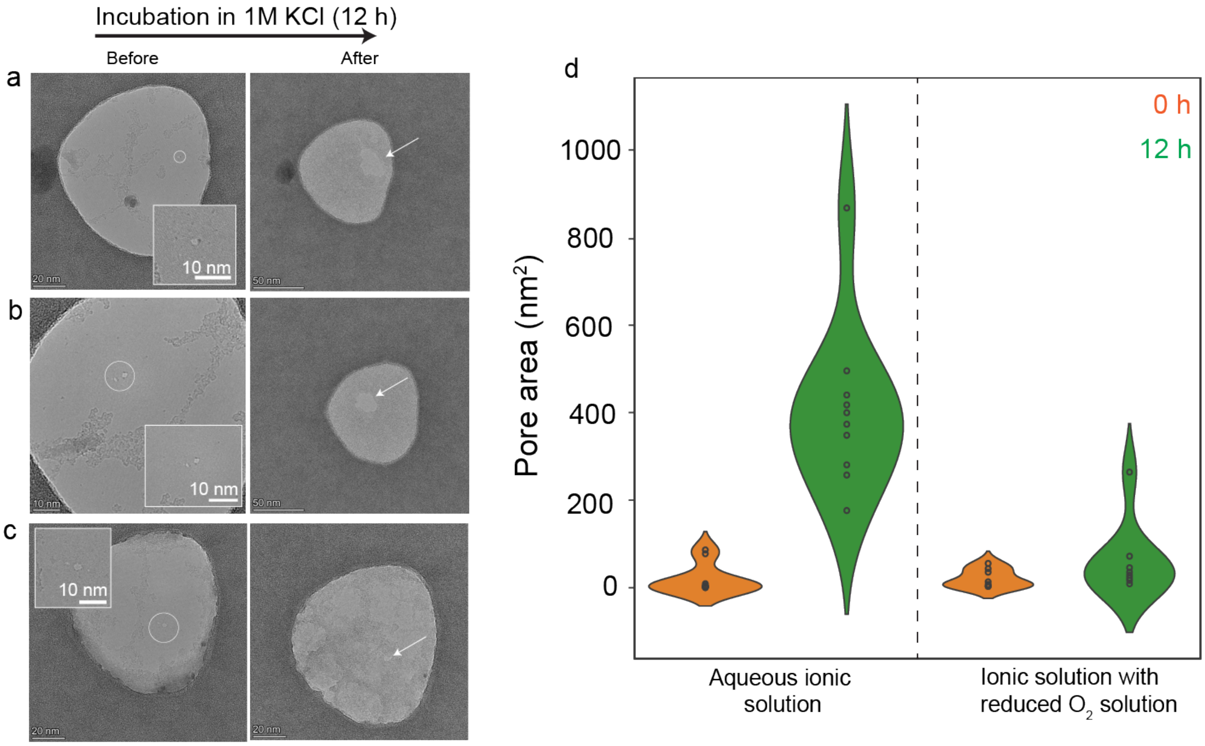

Supplementary Figure 14. TEM images of nanopores before and after incubation in 1M KCl (12 h). (a,b) MoS_2 pores were incubated in a non-degassed buffer with a dissolved O_2 concentration of ~8 mg L^{-1} and (c) in a reduced dissolved O_2 buffer (<1 mg L^{-1}). (d) Violin plots showing the distribution of calculated pore area for different pores before and after expansion in non-degassed (n = 10) and reduced O_2 buffer solution (n = 8). All devices used here are HMDS-coated SiN_x substrates. Note that the violin plots are displayed completely with any truncation at 0. The open-pore area was calculated using Image J.

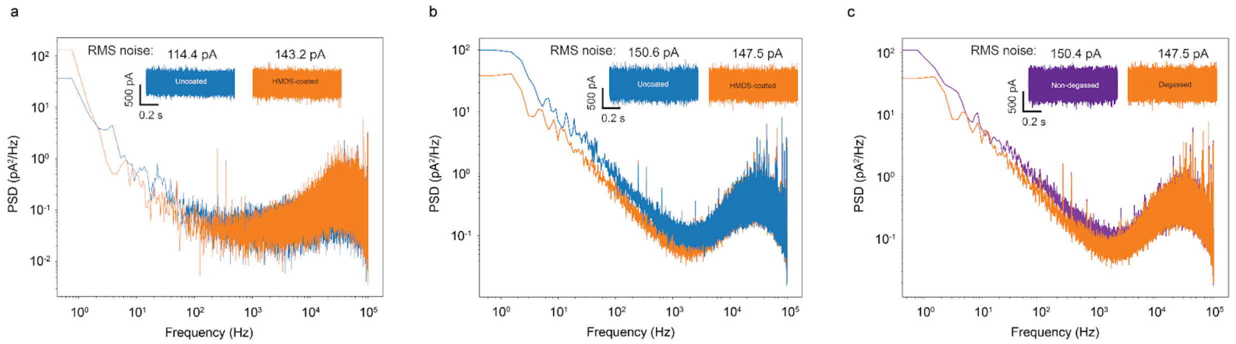

Supplementary Figure 15. Ionic noise comparison of various nanopore devices. (a) Open SiN_x apertures (b) MoS_2 nanopore on uncoated-SiN_x versus HMDS/SiN_x substrate (both devices were ~8 nm in diameter) and (c) MoS_2/HMDS/SiN_x device 1M KCl aqueous buffer (same device) with degassing (dissolved O_2 concentration, 1 mg/L) and without degassing (8 mg/L). The open pore currents in all cases were recorded at 100 mV with 100 kHz filter and 200 kHz sampling rate. We did not observe any significant advantage in terms of noise after coating. The I_{rms} was not significantly different probably due to thin layer of HMDS coating (a). In case of MoS_2 nanopore, the thin membrane dominates the charge fluctuations occurring at the rim of the MoS_2 nanopore as well as the mechanical fluctuations of the thin free-standing 2D membrane contributing additionally to the baseline fluctuations.[2–4]

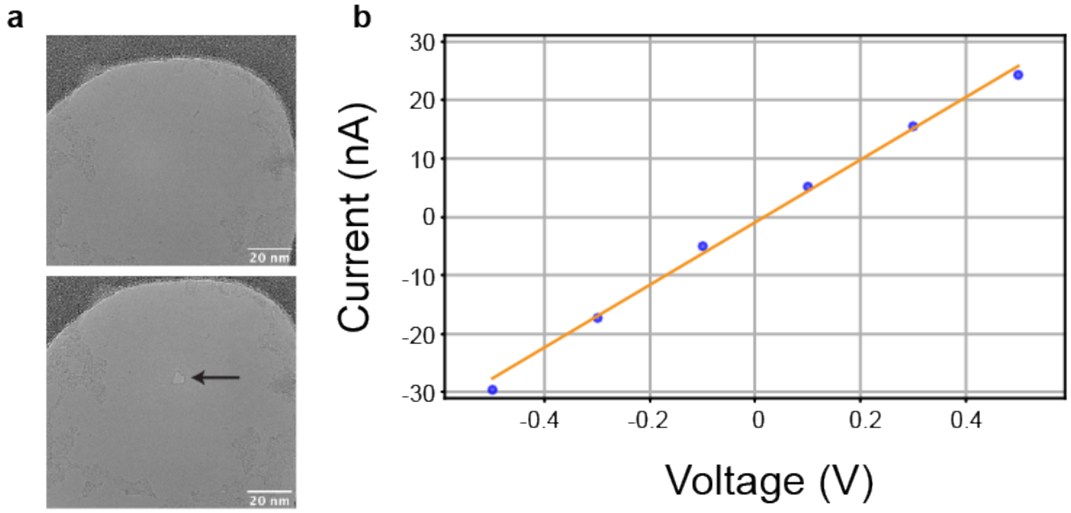

Supplementary Figure 16. (a) Bright-field TEM image of the device used in Figure 6 in the Main text for DNA translocation. The TEM images show free-standing clean monolayer MoS_2 on HMDS/SiN_x substrate before and after drilling a ~6 nm pore using a focused e-beam in TEM at 80 kV. (b) I-V characteristics of the pore (±500 mV) with an open-pore conductance of ~55 nS measured in 1M KCl Tris-EDTA buffer with reduced O_2 concentration (maintained at 1 mg/L).

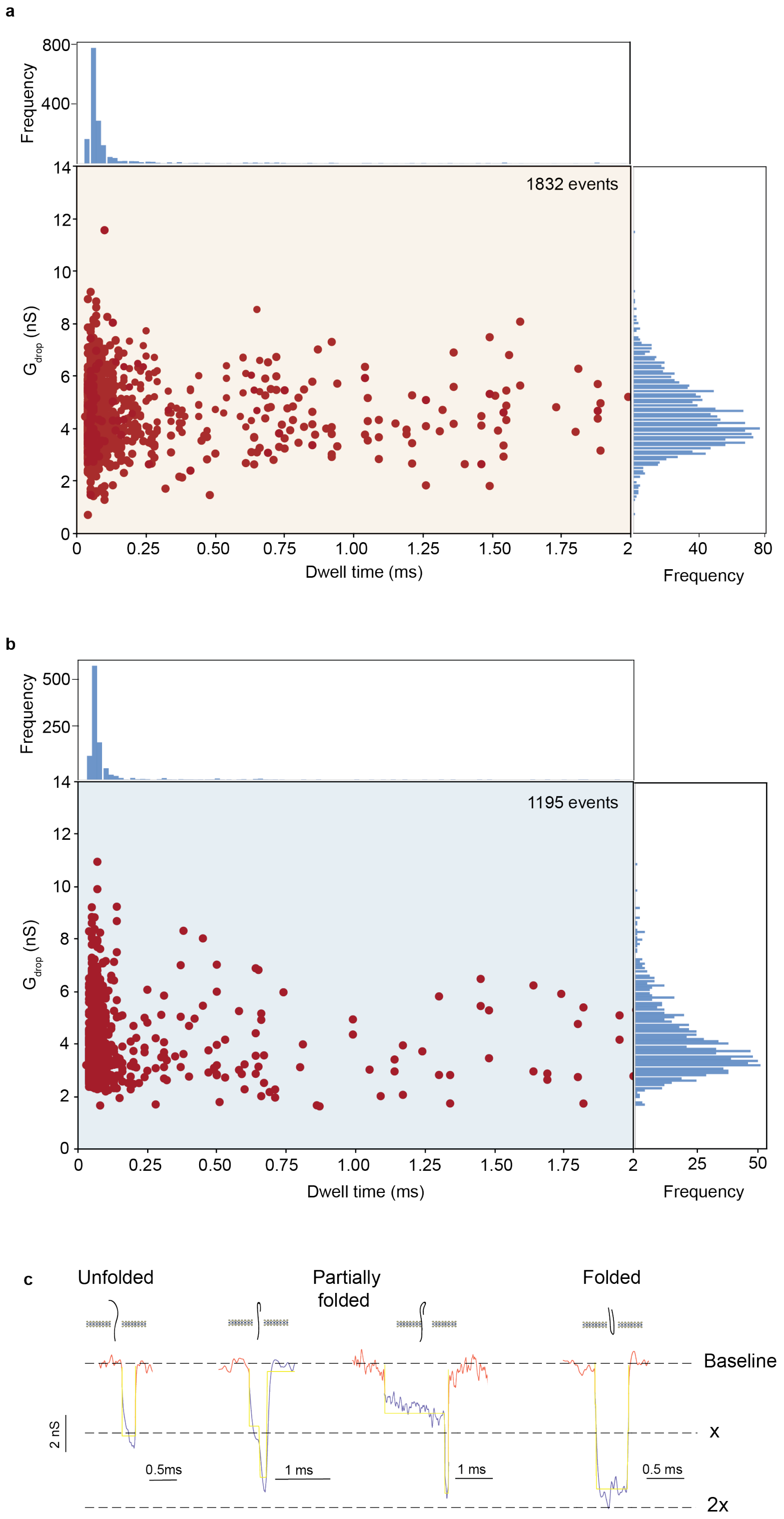
a
Frequency
800
400
14
1832 events
Gdrop (nS)
Dwell time (ms)
Frequency
b
500
250
1195 events
c
Unfolded
Partially folded
Folded
Baseline
x
2x
2 nS
0.5ms
1 ms
1 ms
0.5 ms

Supplementary Figure 17. Double-stranded DNA translocation (1 kbp) through a 6.5 nm MoS_2 nanopore on HMDS/SiN_x substrate from Device used in Figure 6. Scatter plots showing G_{drop} of all the translocation events with dwell time at the (a) beginning of the measurement (0-30 min) and (b) end (150-180 min) of the measurement time at 500 mV. The number of events is shown in the inset. (c) Examples of single dsDNA translocating in different configuration (unfolded, partially folded, and fully folded). The folded configuration typically produces ~2x times of G_{drop} compared to unfolded event as observed before in 2D nanopores[5–7] In partially folded, events (center), increase in dwell time is attributed to sticking of the molecule traversing through the nanopore increasing the dwell time.

Supplementary References:

1. Graf, M. *et al.* Fabrication and practical applications of molybdenum disulfide nanopores. *Nat Protoc* **14**, 1130–1168 (2019).
2. Fragasso, A., Schmid, S. & Dekker, C. Comparing Current Noise in Biological and Solid-State Nanopores. *ACS Nano* **14**, 1338–1349 (2020).
3. Wanunu, M. Nanopores: A journey towards DNA sequencing. *Physics of Life Reviews* **9**, 125–158 (2012).
4. Heerema, S. J. *et al.* 1/f noise in graphene nanopores. *Nanotechnology* **26**, 074001 (2015).
5. Schneider, G. F. *et al.* DNA Translocation through Graphene Nanopores. *Nano Lett.* **10**, 3163–3167 (2010).

6. Danda, G. *et al.* Monolayer WS2 Nanopores for DNA Translocation with Light-Adjustable Sizes. *ACS Nano* **11**, 1937–1945 (2017).
7. Liu, K., Feng, J., Kis, A. & Radenovic, A. Atomically Thin Molybdenum Disulfide Nanopores with High Sensitivity for DNA Translocation. *ACS Nano* **8**, 2504–2511 (2014).
